# Anger instability and aggression in Borderline Personality Disorder – an ecological momentary assessment study

**DOI:** 10.1186/s40479-022-00199-5

**Published:** 2022-10-17

**Authors:** Corinne Neukel, Robin Bullenkamp, Markus Moessner, Karen Spiess, Christian Schmahl, Katja Bertsch, Sabine C. Herpertz

**Affiliations:** 1 Department of General Psychiatry, Medical Faculty, Center for Psychosocial Medicine, Heidelberg University, Heidelberg, Germany; 2 Center for Psychotherapy Research, Heidelberg University, Heidelberg, Germany; 3 Department of Psychosomatic Medicine and Psychotherapy, Medical Faculty Mannheim, Central Institute of Mental Health, Heidelberg University, Heidelberg, Germany; 4 Department of Psychology, LMU Munich, Munich, Germany

**Keywords:** Ambulatory assessment, Anger regulation, Aggressive behavior, Borderline personality disorder, Interpersonal dysfunction

## Abstract

**Background:**

Anger and aggression are core features of Borderline Personality Disorder (BPD), contributing strongly to the individual as well as the societal burden caused by the disorder. Across studies, patients with BPD have shown increased, more frequent and prolonged episodes of anger and reported an increased prevalence of reactive aggression. However, only a few studies have investigated anger and aggression in the patients’ everyday lives and did not consider anger instability. In order to contribute knowledge about aggression and its association with anger intensity and anger instability in real-life in BPD the aim of the present study was to better characterize days with and without aggressive behaviors with regard to the patients’ experienced anger.

**Methods:**

Patients with BPD and high aggression as well as healthy participants took part in an ecological momentary assessment (EMA) study assessing state anger and aggression three times per day over two weeks. Multilevel modeling was conducted and anger instability was operationalized by squared successive differences.

**Results:**

As expected, patients with BPD reported greater instability in their experienced anger compared to healthy participants. Most interestingly, in the BPD group the occurrence of aggressive behavior was significantly associated with anger intensity as well as anger instability. More precisely, on days when patients with BPD acted out aggressively, they reported higher anger intensity as well as greater anger instability than on days when they did not act out aggressively.

**Conclusion:**

Knowledge about what characterizes days with aggressive behaviors may help to improve interventions to reduce aggressive behavior and thus relieve the burden aggression causes for patients with BPD, their surroundings and society.

## Background

Interpersonal deficits are regarded as the most severe and lasting impairments in Borderline Personality Disorder (BPD) [1]. One core symptom of BPD that comes with high costs for the individual as well as for society is inappropriate or intense anger and subsequent aggressive outbursts. Across studies, increased anger intensity, more frequent experiences of anger and prolonged episodes of anger have been shown in patients with BPD: In experimental studies, patients with BPD showed a prolonged anger response after anger induction [2] and a significantly stronger increase in anger compared to healthy participants after a frustration eliciting task [3]. Interestingly, this increase in anger was associated with aggression [3].

In order to assess the intensity and frequency of experienced emotions in real-life, studies started using ecological momentary assessment (EMA), i.e. participants are asked to answer questions about their current emotional state multiple times a day using hand-held devices or smartphones. By this, the experience of emotions can be mapped in the participants’ everyday lives rather than in artificial experimental settings or with cumulative trait questionnaires, adding to ecological validity of the obtained results [4]. In a previous EMA study by Ebner-Priemer and colleagues [5] patients with BPD reported more negative emotions (including anger) and a greater intensity of negative emotions than healthy participants over the course of the day. Using e-diaries, Kockler et al. [6] recently found that specifically anger intensity and frequency is increased in patients with BPD compared to healthy participants and to patients with other mental disorders, underlining the particular importance of anger for patients with BPD. Besides more intense (negative) emotions, affective instability, characterized by rapid and intensive changes in emotional states, is relevant in BPD and constitutes a core feature of the disorder. Such within-person changes in emotional states can be assessed by EMA [4]. Indeed, in previous EMA studies patients with BPD exhibited more rapid changes in emotional states over the day compared to healthy participants [7, 8], however, up to now instability has been investigated for valence of emotional states but not with specific regard to anger.

In BPD, anger often leads to destructive behavior such as aggression. Typically, aggression in BPD is classified as reactive in nature with real or assumed social rejection, threat, provocation, or frustration being assumed to be the most important triggers [3, 9–11]. Previous studies found an increased prevalence of reactive aggression including verbal, but also physical aggression and criminal offending in female and male individuals with BPD [12]. About 73% of patients with BPD report having shown aggressive behavior in the last year [13]. Importantly, aggression is strongly related to anger and difficulties in anger regulation: In females with BPD symptoms anger has been shown to mediate the association between interpersonal rejection and aggression in real-life [11]. Furthermore, trait anger has been associated with aggression in BPD and this association was mediated by emotion dysregulation, i.e., the inability to make use of effective emotion regulation strategies [14].

Despite the disruptive effects on the affected individuals, until now, not much is known about aggression and its association with anger in the patients’ everyday lives. Especially information on what differentiates days when patients act out aggressively from days when they do not is lacking due to the fact that aggression has mainly been studied with trait questionnaires or experimentally in the laboratory environment and not in real-life. Importantly, this information is of clinical relevance as it can contribute to improve and implement psychotherapeutic interventions to prevent aggressive outbursts in affected individuals. Therefore, the main aim of the present study is to better characterize days with and without aggressive behaviors in patients with BPD and high aggression. More precisely, regarding the strong relation between aggression and anger, we aimed at investigating whether anger intensity and changes in the anger experience, i.e. anger instability, differ in patients with BPD on days with and without aggressive behaviors. Since up to now there is no data on instability in anger experience over the day in patients with BPD, we also aimed at comparing patients with BPD and healthy participants with regard to anger instability. To achieve this, we investigated patients with BPD and high aggression as well as healthy participants by means of EMA. Participants were asked three times a day about their state anger and whether they acted out aggressively. We expected (1) anger instability to be more pronounced in patients with BPD than in healthy participants. Regarding our main aim we investigated (2) whether mean reported anger scores are higher on days with aggressive behaviors than on days without aggressive behaviors in patients with BPD and (3) whether instability of experienced anger is higher on days with aggressive behaviors than on days without aggressive behaviors in patients with BPD.

## Methods

### Procedure

This study was part of a project investigating aggression in BPD including a randomized controlled trial (RCT) offering treatment targeting aggression (see [15]). All assessments reported in this study took place before patients with BPD were randomized to one of the treatment groups, healthy participants (HP) did not receive treatment. The RCT was pre-registered in a public trial archive (DRKS00009445) and the study was performed in accordance with the Declaration of Helsinki and approved by the local ethics committee (Faculty of Medicine, Heidelberg University). All participants gave written informed consent before participation. Participants received monetary compensation for taking part in the assessments.

### Participants

Patients with BPD had to meet the following inclusion criteria: age between 18 and 55, at least four BPD criteria according to DSM-IV, thus also including subthreshold BPD, and a score ≥ 6 in the other-directed overt-aggression and irritability subscales of the Modified-Overt Aggression Scale (M-OAS; [16]) over the last two weeks. For all participants reasons of exclusion were pregnancy, substance dependency (except cannabis) or abuse, schizophrenia or bipolar I disorder as well as a change in psychotropic medicine within the last 3 weeks before allocation to the trial. Patients with BPD were recruited via flyers in medical and psychotherapist private practices, press releases in daily newspapers and internet forums as well as via the Department of General Psychiatry at the University Hospital Heidelberg between January 2016 and January 2019 to take part in a randomized controlled trial offering group psychotherapy targeting aggressive behaviors. Before being randomized to and taking part in the intervention they were asked to take part in assessments including behavioral tasks and functional magnetic resonance imaging tasks (reported elsewhere, see [17, 18]) as well as ecological momentary assessments. Additionally, HP were recruited via advertisements in newspapers and internet and letters sent to randomly selected samples of local inhabitants. They were excluded if the criteria of a current or lifetime axis I or axis II disorder were fulfilled, or the overt aggression or irritability subscales of the M-OAS were ≥ 2, thus, this group comprised healthy subjects and not subjects from the general population. The group of HP was matched to the BPD group with regard to age and gender.

Originally 61 patients with BPD and 35 HP were included in the study, of these 55 patients with BPD und 35 HP took part in the EMA. To ensure a sample with adequate compliance, only participants with at least 70% (30 of 42) completed assessments were included, resulting in a sample of *N* = 64 participants (32 BPD and 32 HP).

### Psychometric and clinical assessments

At time of inclusion axis I disorders were assessed by using the Structured Clinical Interview for DSM-IV (SCID-I; [19]) and axis II disorders (BPD, antisocial personality disorder and avoidant personality disorder) were assessed by using the International Personality Disorder Examination (IPDE; [20]). The Modified-Overt Aggression Scale (M-OAS; [16]) was administered to assess overt aggression and irritability within the last two weeks. To measure intelligence (IQ) Raven’s Progressive Matrices [21] were used. All assessments were conducted by trained research diagnosticians (M. Sc. level in clinical psychology or equivalent).

### Ecological momentary assessment

The ecological momentary assessment was conducted over 14 days. All participants received a link to a 14-item questionnaire via an automatic text message on their mobile phone three times a day. The prompts were delivered at a random point of time between (a) 9am and 1pm (b) 1pm and 5pm and (c) 5pm and 9:30pm. If a subject did not complete the questionnaire within 30 min after receiving the link, a reminder message was sent out. The EMA questionnaire was designed to take two to three minutes on average to complete. In the questionnaire the participants had to answer questions about their current state of anger as well as their current emotional arousal and were asked whether they had shown aggressive behavior since the last message. To assess current anger the state-anger subscale of the State Trait Anger Expression Inventory (STAXI-II; [22]) was used. This widely used instrument in anger research [23] measures state anger via 10 items (for example “I’m angry” or “I could blow up with anger”) on a scale of 1 (“not at all”) to 4 (“very”), with a higher score indicating higher state anger. Additionally, aggression was assessed by examining the item “Have you engaged in any verbally or physically aggressive behavior toward people or objects since the last prompt?“ with a dichotomous response scale (“yes” or “no”).

### Statistical analysis

To compare demographic and psychometric data between patients with BPD and HP t-tests were used.

Due to the nested structure of the data (assessments nested within persons), multilevel modeling was conducted. Anger instability was operationalized by squared successive differences (SSD) of EMA assessments within one day. Higher SSD reflect higher instability of anger capturing variability and temporal dependency of the individual’s state anger scores. Although assessments were not perfectly equidistant, adjusted SSD (ASSD) were highly correlated to SSD and yielded similar results. As proposed by Jahng et al. [24], to test differences in instability gamma models with log link functions and random intercepts were applied (glmer command in lme4 package; [25]). Differences in average anger levels between days with and without behavioral aggression were tested with mixed effect models with random intercepts (lme command in nlme package; [26]). Research questions 2) and 3) concerning days with and without aggressive behaviors were investigated only in the group of patients with BPD.

All analyses were conducted using R (R Development Core Team, 2011).

## Results

### Sample characteristics

Four patients from the BPD group met four BPD criteria according to DSM-IV, the rest of the patients met five or more criteria. The BPD and the HP group did not differ with regard to age and gender, but differed with regard to IQ, while in both groups the mean IQ is in the range of one standard deviation (see Table 1). For comorbid disorders in the BPD group please refer to Table 1. Patients with BPD showed an average of 87.8% completed assessments and HP of 90.4%. In all, 164 days with and 280 days without aggressive behaviors were reported in the BPD group, while 6 patients with BPD did not report any aggressive behavior during the survey period. In the HP group, 5 days with and 443 days without aggressive behaviors were reported.

**Table 1:** Sample description

	BPD	HP	T	p
N Gender (female)	32	32		
	23 (72%)	23 (72%)		
Age (Years)	32.8 ± 10.1	30.3 ± 7.2	1.14	0.259
Intelligence (Raven IQ)	105.2 ± 10.2	112.7 ± 11.1	2.78	0.007
Compliance (%)	87.8	90.4	1.37	0.174
Axis I comorbidity (N)	current (lifetime)			
Affective disorder	18 (8)	-		
Substance dependency	7 (0)	-		
Anxiety disorder	14 (14)	-		
Posttraumatic Stress Disorder	8 (10)	-		
Eating Disorder	7 (3)	-		
Axis II comorbidity (N)				
Antisocial personality disorder	2 (1)	-		
Avoidant personality disorder	5 (5)	-		

Notes. BPD: Borderline Personality Disorder; HP: healthy participants

### Anger intensity and anger instability.

Table 2 displays the results for the first and Table 3 for the second and third hypotheses.

Overall, the BPD group displayed higher levels of anger instability than healthy participants (p < .001). The associations of anger intensity and anger instability with aggression were investigated in the BPD group only. Participants in the BPD group displayed significantly higher anger scores on days with aggressive behaviors (p < .001). On average, anger scores were elevated by more than four points. In addition, participants in the BPD group also displayed higher anger instability on days with aggressive behavior (p < .001).

**Table 2:** Results of the mixed effects models for hypothesis 1

	Fixed Effects			
	Estimate	SE	t	p
SSD* (n = 1,451)				
Intercept	-0.106	0.368	-0.289	0.77
Group (1 = BPD)	4.110	0.520	7.901	< 0.001

Notes. *Gamma link function; SSD: squared successive differences; BPD: Borderline Personality Disorder

**Table 3:** Results of the mixed effects models for hypotheses 2 and 3

	Fixed Effects			
	Estimate	SE	t	p
Anger (n = 1,179)				
Intercept	15.699	0.453	34.650	< 0.001
Aggression day (1 = YES)	4.646	0.452	10.282	< 0.001
SSD* (n = 699)				
Intercept	3.563	0.165	21.569	< 0.001
Aggression day (1 = YES)	0.965	0.189	5.118	< 0.001

Notes. *Gamma link function; SSD: squared successive differences; BPD: Borderline Personality Disorder

## Discussion

This is one of the first studies that investigated anger and aggression via EMA in the everyday lives of patients with BPD and HP. We found that patients with BPD compared to HP report greater instability in their experienced anger. Most importantly, we were able to better characterize days with aggressive behaviors in patients with BPD: The occurrence of aggressive behavior was significantly related to mean anger intensity as well as anger instability. On days when patients acted out aggressively, they reported higher mean anger intensity as well as greater anger instability than on days when they did not act out aggressively.

Confirming our first hypothesis, the present result of greater instability of experienced anger in patients with BPD compared to HP is in line with previous results of increased affective instability in patients with BPD [8]. The result highlights that patients with BPD are able to perceive variability of their angry feelings and that anger is not perceived as constantly high as might be assumed regarding the increased levels of state and trait anger assessed with self-report questionnaires and in experimental settings [3, 27].

Most interestingly, the present results confirm the association between anger and aggression as previously shown using trait questionnaires and in experimental settings in the everyday lives of patients with BPD. In line with our second and third hypothesis, the occurrence of aggressive behavior in the present sample is not only related to the mean anger intensity of the day, but also to anger instability. Trait anger intensity and anger increase in the experimental setting have previously been shown to be associated with aggression [3, 14]. In addition, anger instability may contribute to the emergence of aggressive behavior, possibly by making it more difficult for patients to use strategies of affect regulation. Although the current literature strongly points to the specific role anger plays with regard to aggressive behavior, based on our current data it is not yet clear whether the association between anger instability and aggression is specific for anger or whether aggression might be associated with affective instability in general.

The present data are the first to indicate that patients with BPD report different anger intensity as well as anger instability on days when they also report to have acted out aggressively compared to days when they did not show aggressive behaviors. Knowledge about what characterizes days when patients act out aggressively can contribute to the improvement of psychotherapeutic interventions and skills to prevent aggressive outbursts. We have recently developed a mechanism-based anti-aggression psychotherapy for patients with BPD that encompasses elements from dialectical behavior therapy as well as mentalization based therapy and has been shown to be effective in reducing anger and aggression in a proof-of-concept study [15]. The significance of anger instability in characterizing days with aggressive outbursts underlines the importance of training patients to monitor their emotions. For instance, instability of anger experience over the day may serve as a warning signal to patients that they might be prone to show aggressive behavior and may thus prompt them to use a specific skill. Especially ecological momentary interventions that offer real-time and context-aware interventions in the patients’ everyday lives [28] will be important tools complementing psychotherapy.

Importantly, our results indicate that intensity and instability of anger are both associated with the emergence of aggressive behavior in BPD. However, while the current results add to our knowledge of what characterizes days with aggression, they do not allow conclusions on the exact process that leads from anger to aggression. Hence, another explanation of the result regarding the association between occurrence of aggression and anger instability might be that aggressive behavior reduces intense feelings of anger as suggested by the Emotional Cascade Model [29]. Besides, an elevated tendency in patients with BPD to ruminate on anger might contribute to increased anger intensity and instability, and has previously been shown to enhance the risk for aggressive behaviors [30]. Furthermore, not much is known about situational triggers of anger and subsequent aggression in real-life, a question that should be addressed in future studies.

We would like to acknowledge some limitations of the present study. First, results should be replicated in a bigger sample. Second, the participants in the present study only received prompts for three assessments per day. To further improve the assessment of fluctuating affective states and to be able to map the process leading from anger to aggression, future studies should encompass more frequent assessments per day while keeping in mind the burden for the participants. Lastly, since the present study does not include a clinical control group, it cannot be concluded that greater anger instability is specific for patients with BPD. Future studies should include groups of patients prone to aggressive behavior with different mental disorders, such as patients with bipolar disorder, to investigate whether anger instability of patients with BPD differ from these, too.

## Conclusion

Despite the named limitations, the present study represents an important step to better understand aggression in the everyday lives of patients with BPD, a feature of high clinical relevance in BPD. Going beyond trait questionnaires and experimental paradigms it confirms the association between anger intensity and aggression in real-life and shows that additionally anger instability seems be associated with aggression in real-life, too. This not only adds to our understanding of aggression in BPD but can also be used to improve interventions preventing aggressive behavior and by this relieve patients with BPD and their surroundings.

## Data Availability

The datasets for this study are available on request from the corresponding author.

## Abbreviations

BPD: Borderline Personality Disorder.
EMA: Ecological Momentary Assessment.
RCT: randomized controlled trial.
HP: healthy participants.
M-OAS: Modified-Overt Aggression Scale.
SCID-I: Structured Clinical Interview for DSM-IV.
IPDE: International Personality Disorder Examination.
STAXI-II: State Trait Anger Expression Inventory.
SSD: squared successive differences.
ASSD: adjusted squared successive differences.
